# Free Vibration Analysis of a Graphene-Reinforced Porous Composite Plate with Different Boundary Conditions

**DOI:** 10.3390/ma14143879

**Published:** 2021-07-12

**Authors:** Hong-Gang Pan, Yun-Shi Wu, Jian-Nan Zhou, Yan-Ming Fu, Xin Liang, Tian-Yu Zhao

**Affiliations:** 1 Shenyang Institute of Engineering, School of Energy and Power, Shenyang 110136, China; phg666@163.com (H.-G.P.); Wuys0621@163.com (Y.-S.W.); lx18340158686@163.com (X.L.); 2 Technology Center of Shenyang Customs, Shenyang 110016, China; tank261014@163.com; 3 Laboratory Management Center, Shenyang Sport University, Shenyang 110102, China; 4 School of Science, Northeastern University, Shenyang 110819, China

**Keywords:** graphene platelets, free vibration frequency, porosity, boundary conditions

## Abstract

Plates are commonly used in many engineering disciplines, including aerospace. With the continuous improvement in the capacity of high value-added airplanes, large transport aircrafts, and fighter planes that have high strength, high toughness, and corrosion resistance have gradually become the development direction of airplane plate structure production and research. The strength and stability of metal plate structures can be improved by adding reinforced materials. This paper studies graphene platelets (GPLs) reinforced with a free vibration porous composite plate. The porous plate is constructed with a multi-layer model in a metal matrix containing uniform or non-uniformly distributed open-cell internal pores. Considering the random and directional arrangement of graphene platelets in the matrix, the elastic modulus of graphene composites was estimated using the Halpin–Tsai micromechanical model, and the vibration frequencies of graphene composite were calculated using the differential quadrature method. The effects of the total number of layers, GPL distribution pattern, porosity coefficient, GPL weight fraction, and boundary conditions on the free vibration frequency of GPLs reinforced porous composite plates are studied, and the accuracy of the conclusions are verified by the finite element software.

## 1. Introduction

Graphene is a material with one of the highest known strengths. The theoretical Young’s modulus of graphene is up to 1.0 TPa and it has an inherent tensile strength of 130 GPa. The morphology of graphene is similar to the lamellar structure of thin paper and the thickness of a single layer is only 0.335 nm. It is the thinnest two-dimensional material presently known. Furthermore, it has good toughness, reproducibility, high dispersibility, and good chemical and biocompatibility [1]; thus, it has become an ideal reinforcement for composite materials. Additionally, a composite plate is made from a metal matrix plate, as a continuous phase, and has a sized graphene modifier as a dispersed phase. The modifier is dispersed in the matrix material through appropriate preparation methods in order to form a composite system that contains sized materials. Graphene reinforced porous composite plates have a lighter weight, higher stiffness, higher strength, and multi-functional properties that can meet the lightweight requirements of the aerospace field and inject new vitality into the development of aerospace materials.

Scholars have been paying more and more attention to the study of free vibration graphene reinforced porous composite plates. Rahman et al. [2] used molecular mechanics and molecular dynamic simulations to study graphene epoxy resin-based composites. The results showed that adding graphene platelet (GPL) to the epoxy resin matrix could significantly improve the Young’s modulus and shear modulus of the matrix materials. King et al. [3] found that carbon-filled epoxy composites have high specific stiffness, which can be used for the structural components of fixed-wing aircrafts. Feng et al. [4] studied the vibration analysis of multi-layer graphene sheets using a continuum model. This paper confirms the use of the GNP aspect ratio and the two-dimensional randomly oriented filler Halpin–Tsai model, adjusted for platelet filler shapes, to predict the tensile modulus well for the GNP/epoxy composites, which provides a basic theory for our subsequent research. Alibeigloo [5] used non-local continuum mechanics to conduct a three-dimensional vibration analysis of multi-layer graphene platelets embedded in a polymer matrix. The numerical results show that the bending properties can be significantly improved by adding a small amount of GPLs into the polymer matrix. Yang et al. [6] studied the nonlinear bending and buckling behavior of graphene reinforced nanocomposite beams, based on the Timoshenko beam theory, and the results showed that composite beams doped with graphene platelets could exhibit better mechanical properties. Eringen [7,8,9], by the use of nonlocal thermodynamics and invariance under rigid motions, obtained constitutive equations for the nonlinear micromorphic elastic solids and differential equations of nonlocal elasticity and solutions for screw dislocation and surface waves. Polit et al. [10] analyzed the bending and elastic stability of thick beams with a hole based on the high-order shear deformation theory of the transverse tensile effect. The formula in this paper provides an important reference for the writing of this paper. Rafiee et al. [11] experimentally found that GPLs has the distinct advantage of reinforcing nanofillers over carbon nanotubes at a very low content, which has also been theoretically confirmed [12,13,14,15]. Zhao et al. [16] studied the rubbing of the mistuned bladed disk system with blades of variable thicknesses, and elastically supported shaft-variable thickness blades coupled with the finite element model was established. Sharma et al. [17,18] studied the reduction of sound pressure at the receiver location with a lumped mass at the optimal location, which was shown to be much more than what is achievable by a uniform distribution of the point mass over the plate. A novel concept of the local mitigation of the transmitted noise at a target receiver location is presented by controlling the directivity of the transmitted noise through a point mass attachment on the barrier surface. Zhao et al. [19,20,21] findings shed an important light on the design of the novel graphene reinforced blade-shaft system and remarkably improved its dynamic performance. Sharma et al. [22] studied the effect of uncertainties in material and geometric parameters on the acoustic performance of a viscoelastic coating. Zhao et al. [23,24,25] investigated the free vibration behaviors of a functionally graded (FG) disk-shaft rotor system, which was reinforced with a graphene nanoplatelet (GPL) that rested on elastic supports. Shafiei et al. [26] studied the size-dependent nonlinear vibration behavior of FG porous microbeams using the improved coupling of the stress and Euler–Bernoulli theory, and evaluated the effects of uniform and non-uniform porosity. Sharma et al. [27] studied the effect of strong and weak coupling of void resonances on the transmission characteristics and drew the conclusion that strong coupling of the resonance of voids results in broadband attenuation of sound. Davletshin et al. [28] found that an interlayer distance change leads to significant band gap size modulations and direct–indirect band gap transitions in the phosphorene–BN heterostructure. Babicheva et al. [29] found that a less dense structure may actually be stronger due to the fact that all the interatomic bonds in it are loaded more uniformly. Savin et al. [30] found that the thermal conductivity coefficient of the nanoribbon increases monotonically up to 10%, with an increasing twist angle; the regime of uniform twisting and twist deformation of nanoribbons can improve their mechanical and physical properties. Savin et al. [31] revealed that layered materials can support surface ripplocations that are highly mobile, topologically solitary waves that efficiently transport mass and energy. Chen et al. [32] conducted a numerical study on the crushing process of an FG porous structure, and pointed out that, under high-speed impact, a certain type of non-uniform asymmetric pore can significantly promote the energy absorption of foam metal. The above research results are very important and provide the basis for subsequent research. Before the porous composites can be applied to engineering applications, a large amount of corresponding research work is still needed in order to reveal the structural properties of porous composites.

In this paper, graphene platelets were mixed into copper-based, porous square plates to enhance the material properties of square copper-based plates. The study on the combination of a porous structure and graphene reinforced composites will better improve the performance of the copper substrate, and provide a reference for the preparation of a high performance graphene copper matrix composite material that is light weight, has a high modulus and high strength in the future. The Young’s modulus and shear modulus of the porous graphene composite plate were calculated using the Halpin–Tsai micromechanical model, and the vibration equation of the plate was established. The free vibration frequencies under four boundary conditions were solved by the calculation. The effects of porous distribution, the distribution mode, content and geometric size of the free vibration frequency of the porous graphene composite plate were analyzed.

## 2. Material Properties Calculation and Model Establishment

**Figure 1:**
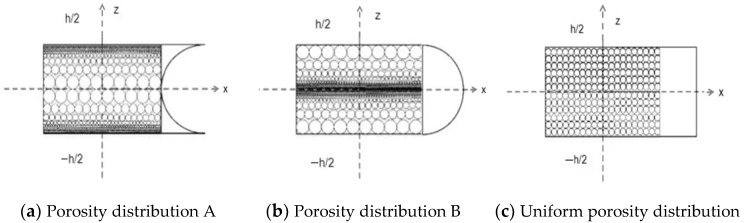
Three types of porosity distributions.

**Figure 2:**
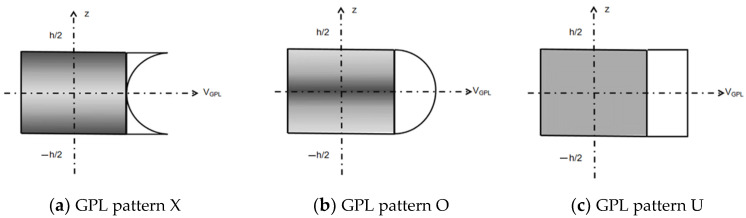
Three types of GPL dispersion pattern.

The three pore distributions in the porous plate include two non-uniform symmetric pore distributions and one uniform pore distribution, as shown in Figure 1. The maximum pore size of the non-uniform distribution mode A is distributed on the middle plane, and the maximum pore size of the non-uniform distribution mode B is distributed on the top and bottom surfaces, which correspond to the changes in material properties and to Equations (1) and (2). The material properties of uniformly distributed pores correspond Equation (3). Meanwhile, the three distribution modes (X, O, and U) of the GPL dispersion pattern are shown in Figure 2.

Non-uniform porosity distribution A:

(1) $$\begin{array}{l} E(z)=E^{*}[1-e_{0}\cos(\pi z/h)]\\ G(z)=G^{*}[1-e_{0}\cos(\pi z/h)]\\ \rho (z)=\rho^{*}[1-e_{m}\cos(\pi z/h)] \end{array}$$

Non-uniform porosity distribution B:

(2) $$\begin{array}{l} E(z)=E^{*}[1-e_{0}^{*}(1-\cos(\pi z/h))]\\ G(z)=G^{*}[1-e_{0}^{*}(1-\cos(\pi z/h))]\\ \rho (z)=\rho^{*}[1-e_{m}^{*}(1-\cos(\pi z/h))] \end{array}$$

Uniform porosity distribution:

(3) $$\begin{array}{l} E(z)=E^{*}\alpha \\ G(z)=G^{*}\alpha \\ \rho (z)=\rho^{*}\alpha^{\prime } \end{array}$$

where *ρ*(*z*), *G*(*z*), and *E*(*z*) are the mass density, shear modulus, and Young’s modulus of the porous composite plates, ρ^*^, G^*^, and E^*^ are the corresponding properties of the GPLs reinforced porous composite plates without internal pores, *e*_0_ and $e_{0}^{*}$ (0 ≤ *e*_0_($e_{0}^{*}$) < 1) in *G*(*z*) and *E*(*z*) are the porosity coefficients for distributions A and B, *e_m_* and $e_{m}^{*}$ in *ρ*(*z*) are the corresponding coefficients of the mass densities, and α and α’ are the parameters for the uniform porosity.

Based on Halpin–Tsai micromechanical model [33], the Young ‘s modulus E^*^ of graphene nonporous composites was determined:

(4) $$E^{*}=\frac{3}{8}\left(\frac{1+\xi_{L}^{GPL}\eta_{L}^{GPL}V_{GPL}}{1-\eta_{L}^{GPL}V_{GPL}}\right)E_{m}+\frac{5}{8}\left(\frac{1+\xi_{W}^{\mathrm{GPL}}\eta_{W}^{GPL}V_{GPL}}{1-\eta_{W}^{GPL}V_{GPL}}\right)E_{m}$$

In which:

$$\begin{array}{l} \xi_{L}^{GPL}=\frac{2L_{\mathrm{GPL}}}{T_{GPL}}\\ \xi_{W}^{GPL}=\frac{2W_{GPL}}{T_{GPL}}\\ \eta_{L}^{GPL}=\frac{E_{GPL}-E_{m}}{E_{GPL}+\xi_{L}^{GPL}E_{m}}\\ \eta_{W}^{GPL}=\frac{E_{GPL}-E_{m}}{E_{GPL}+\xi_{W}^{GPL}E_{m}} \end{array}$$

where *E_GPL_* and *E_m_* are the Young’ s modulus of graphene and the metal matrix in the composites. The metal matrix material used in this paper was copper, *L_GPL_*, *W_GPL_* and *T_GPL_* are the length, width, and thicknesses of the graphene platelets, and *V_GPL_* is the volume content of graphene. The density and Poisson’s ratio of the composites were obtained according to the extended rule of the mixture [34]:

(5) $$\begin{array}{l} \rho^{*}=\rho_{GPL}V_{GPL}+\rho_{m}\left(1-V_{GPL}\right)\\ \upsilon^{*}=\upsilon_{GPL}V_{GPL}+\upsilon_{m}\left(1-V_{GPL}\right) \end{array}$$

where ρ^*^ and υ^*^ are the mass density and Poisson s ratio of GPLs, and *ρ_m_* and *υ_m_* are the corresponding parameters of the metal matrix. The Poisson’s ratio for open metal foams was fixed. The shear modulus G^*^ of the composite plate was:

(6) $$G^{*}=\frac{E^{*}}{2(1+\upsilon^{*})}$$

The typical mechanical properties of open-cell metal foams, shown in Equation (7), were employed to establish the relationships, in Equation (8), between the mass density coefficients and the porosity coefficients for different porosity distributions.

(7) $$\frac{E\left(z\right)}{E^{*}}={\left(\frac{\rho (z)}{\rho^{*}}\right)}^{2}$$

(8) $$\begin{array}{l} 1-e_{m}\cos\left(\pi z/h\right)=\sqrt{1-e_{0}\cos\left(\pi z/h\right)}\\ 1-e_{m}^{*}\left(1-\cos\left(\pi z/h\right)\right)=\sqrt{1-\cos\left(\pi z/h\right)}\\ \alpha^{\prime }=\sqrt{\alpha } \end{array}$$

The mass and graphene distribution of porous composite plates with different porosity were considered to be equivalent. The formula is as follows:

(9) $$\begin{array}{l} \int_{0}^{h/2}\sqrt{1-e_{0}^{*}\left(1-\cos\left(\pi z/h\right)\right)}dz=\int_{0}^{h/2}\sqrt{1-e_{0}\cos\left(\pi z/h\right)}dz\\ \int_{0}^{h/2}\sqrt{\alpha }dz=\int_{0}^{h/2}\sqrt{1-e_{0}\cos\left(\pi z/h\right)}dz \end{array}$$

Formula (9) can determine $e_{0}^{*}$ and α by giving the value of *e*_0_ [35], as shown in Table 1.

**Figure 3:**
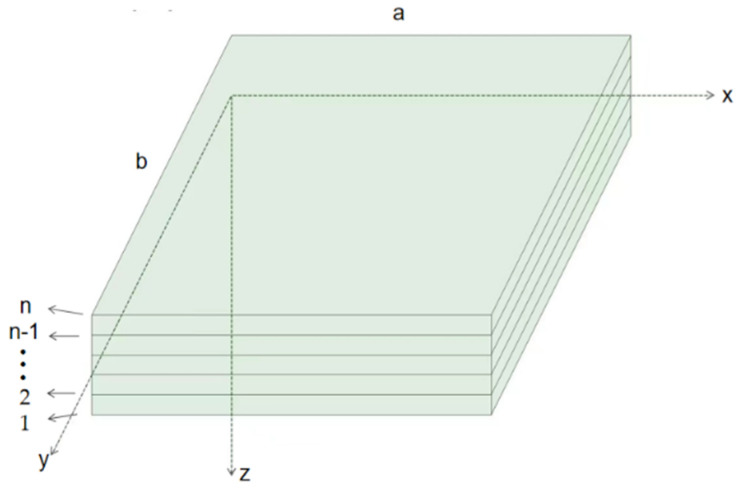
Configuration of a multi-layer plate model.

The 3D model of the multilayer thin plate (length a = 1 m, width b = 1 m, thickness h = 0.02 m) was established and defined in the Cartesian coordinate system (x, y, z), as shown in Figure 3. Each layer had the same thickness, and the internal pores and graphene were evenly distributed. Based on the following convergence analysis, the optimal total number of layers (n) was discussed and determined.

For different distribution patterns, the volume content V*_GPL_* was the graphene changes along the thickness direction.

(10) $$V_{GPL}(z)=\left\{ \begin{array}{l} s_{i1}[1-\cos(\pi z/h)]\\ s_{i2}\cos(\pi z/h)\\ s_{i3} \end{array}\right.\begin{array}{l} GPL_{}pattern_{}X\\ GPL_{}pattern_{}O\\ GPL_{}pattern_{}U \end{array}$$

where *s_i_*_1_, *s_i_*_2,_ and *s*_i3_ are the maximum values of the volume content, and *i* = 1, 2, 3 corresponding to the porosity distributions A and B and the uniform distribution. The total GPL volume content $V_{GPL}^{T}$ was calculated from the weight fraction ∧*_GPL_* in Equation (11), and was then used to determine *s_i_*_1_, *s_i_*_2_ and *s_i_*_3_ in Equation (12), with the aid of the multi-layer plate model.

(11) $$V_{GPL}^{T}=\frac{\Lambda_{GPL}\rho_{m}}{\Lambda_{GPL}\rho_{m}+\rho_{GPL}-\Lambda_{GPL}\rho_{GPL}}$$

(12) $$V_{GPL}^{T}\sum_{j=1}^{n}\frac{\rho (z_{j})}{\rho^{*}}=\left\{ \begin{array}{l} s_{i1}\sum_{j=1}^{n}\left\{[1-\cos(\pi z_{j}/h)]\frac{\rho (z_{j})}{\rho^{*}}\right\}\\ s_{i2}\sum_{j=1}^{n}\left\{\cos(\pi z_{j}/h)]\frac{\rho (z_{j})}{\rho^{*}}\right\}\\ s_{i3}\sum_{j=1}^{n}\frac{\rho (z_{j})}{\rho^{*}} \end{array}\right.$$

In which: $z_{j}=\left(\frac{1}{2}+\frac{1}{2n}-\frac{j}{n}\right)h,j=1,2,3,\ldots ,n$.

## 3. Differential Equation of Thin Plate Vibration

The thin plate was an ideal mechanical model that satisfied certain assumptions. Generally, a certain actual problem is simplified on a thin plate model according to its actual size and force characteristics. For example, a thin plate model can be used for a structure with a thickness smaller than its length and width. According to the coordinate system in Figure 3, the relationship between stress, strain, and displacement is established. Set the position of any point a on the board and determine its coordinates x, y, and z before deformation. According to the classical theory of thin plates, the expression of the displacement components of any point a (x, y, and z) along the three directions can be obtained as:

(13) $$\begin{array}{l} u_{a}=-z\frac{\partial w}{\partial x}\\ \nu_{a}=-z\frac{\partial w}{\partial y}\\ w_{a}=w \end{array}$$

According to the geometric relationship between strain and displacement, the three main strain components at each point can be calculated as:

(14) $$\begin{array}{l} \varepsilon_{x}=\frac{\partial u_{a}}{\partial x}=-z\frac{\partial^{2}w}{\partial x^{2}}\\ \varepsilon_{y}=\frac{\partial \nu_{a}}{\partial y}=-z\frac{\partial^{2}w}{\partial y^{2}}\\ \gamma_{xy}=\frac{\partial u_{a}}{\partial y}+\frac{\partial \nu_{a}}{\partial x}=-2z\frac{\partial^{2}w}{\partial x\partial y} \end{array}$$

Use Hooke’s law to obtain the corresponding three main stress components as:

(15) $$\begin{array}{l} \sigma_{x}=\frac{E}{1-\mu^{2}}(\varepsilon_{x}+\mu \varepsilon_{y})=-\frac{Ez}{1-\mu^{2}}(\frac{\partial^{2}w}{\partial x^{2}}+\mu \frac{\partial^{2}w}{\partial y^{2}})\\ \sigma_{y}=\frac{E}{1-\mu^{2}}(\varepsilon_{y}+\mu \varepsilon_{x})=\frac{Ez}{1-\mu^{2}}(\frac{\partial^{2}w}{\partial y^{2}}+\mu \frac{\partial^{2}w}{\partial x^{2}})\\ \tau_{xy}=G\gamma_{xy}=-\frac{Ez}{1+\mu }\frac{\partial^{2}w}{\partial x\partial y} \end{array}$$

According to Σ*F_z_* = 0, Σ*M_x_* = 0, and Σ*M_y_* = 0, in which Σ*F_z_* is the resultant force in the z direction and Σ*M_x_* and Σ*M_y_* are the resulting moments in the x and y direction.

(16) $$\begin{array}{l} Q_{x}dy+\frac{\partial Q_{y}}{\partial x}dydx-Q_{x}dy+Q_{y}dx+\frac{\partial Q_{x}}{\partial y}dxdy-Q_{y}dx\\ +P(x,y)f(t)dydx-\rho h\frac{\partial^{2}w}{\partial t^{2}}dydx=0\\ (M_{x}dy+\frac{\partial M_{x}}{\partial x}dxdy)-M_{x}dy+(M_{yx}dx+\frac{\partial M_{yx}}{\partial y}dxdy)-M_{yx}dx\\ -(Q_{x}dy+\frac{\partial Q_{x}}{\partial x}dxdy)\cdot \frac{1}{2}dx-Q_{x}dy\cdot \frac{1}{2}dx=0\\ M_{y}dx-(M_{y}dx+\frac{\partial M_{y}}{\partial y}dydx)+\frac{1}{2}Qdxdy(Q_{y}dx+\frac{\partial Q_{Y}}{\partial y}dydx)\\ -M_{xy}dy-(M_{xy}dy+\frac{\partial M_{xy}}{\partial x}dxdy)=0 \end{array}$$

*Q_x_* and *Q_y_* are the shear stresses in the x and y direction, *P* (x, y, t) =*P* (x, y) *f* (t) is the external load degree set along the z axis with a variable separation form. Reorganize the above formula in order to obtain the differential equation of the lateral free vibration of the rectangular plate:

(17) $$D[\frac{\partial^{4}w}{\partial x^{4}}+2\frac{\partial^{4}w}{\partial x^{2}\partial y^{2}}+\frac{\partial^{4}w}{\partial y^{4}}]+\rho h\frac{\partial^{2}w}{\partial t^{2}}=0$$

*D* is the bending stiffness of the thin plate. The form of the solution is that the time variable and the coordinate variable can be separated:

(18) w(x,y,t)=W(x,y)cosωt

*w* (*x*, *y*, and *t*) is the transverse displacement, and the following can be obtained:

(19) $$\nabla^{4}W-k^{4}W=0$$

where: $k^{4}=\frac{\rho h}{D}\omega^{2}$.

The boundary conditions studied in this paper include fixed-supported edge and simply-supported edge, and their forms are:

(20) $$w=0,M_{n}=0\;\mathrm{Simple-support}\;\mathrm{edge}\;\mathrm{is}\;\mathrm{represented}\;\mathrm{by}\;S$$

(21) $$w=0,\frac{\partial w}{\partial n}=0\;\mathrm{The}\;\mathrm{fixed}\;\mathrm{edge}\;\mathrm{is}\;\mathrm{represented}\;\mathrm{by}\;C$$

## 4. Finite Element Analysis

The free vibration frequency of the porous composite plates can be calculated using the above formula. The MATLAB 2018 software and ANSYS 19.0 software were used for analysis in this study. MATLAB integrates many powerful functions, such as numerical analysis, matrix calculation, scientific data visualization, and modeling and simulating nonlinear dynamic systems into an easy-to-use window environment. MATLAB contains a large set of computing algorithms. It has more than 600 mathematical operation functions used in engineering, which can easily realize the various calculation functions required by users. The algorithms used in the function are the latest research results in scientific research and engineering calculations and have been optimized and made fault tolerant. MATLAB programming of the formula in the third section into the software can make the calculation results more accurate and efficient. ANSYS software is a large general finite element analysis software that uses the mathematical approximation method to simulate the real physical system (geometry and load conditions), and the analysis results of ANSYS simulation software are more suitable for engineering practice. The ANSYS and MATLAB calculation method is different but can calculate the free vibration frequency and vibration pattern. The ANSYS software was used to analyze the vibration frequency and vibration pattern of the composite plate. The results from the MATLAB software and ANSYS software under the same conditions were compared. If the error was less than 5%, it indicated that the calculation of the graphene reinforced porous composite plate in this paper was correct, and the influence of other factors on the vibration frequency of the plate can be further discussed. Because the boundary conditions of plates in this paper only consider the clamped and simply-supported edges, we choose four classical boundary conditions: SSSS, SSCC, SCCC, and CCCC. From the results of these four boundary conditions, we can find the influence of boundary conditions on plate vibration frequency. The following data are the first four-order analysis results and graphical comparisons of MATLAB and ANSYS with the boundary conditions: SSSS, SSCC, SCCC, CCCC, Porosity distribution A, and GPL pattern X.

Observation Table 2 shows that, under different boundary conditions of the porous composite plate, the difference between the four-order free vibration frequencies calculated by MATLAB and ANSYS was below 5%, which verified the accuracy of the calculated results. The ANSYS and MATLAB results of the first four vibration modes of the graphene-reinforced porous composite plate with the boundary conditions of SSSS, Porosity distribution A, and GPL pattern X are shown in the following figure.

**Figure 4:**
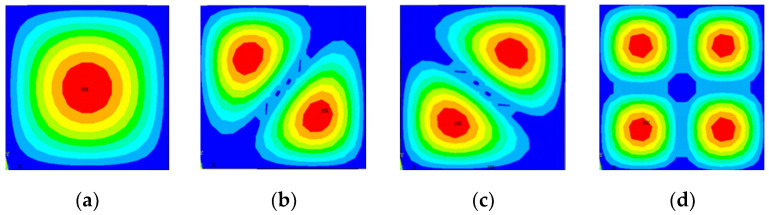
The first four-order vibration mode of ANSYS. (**a**) First order vibration mode, (**b**) Second order vibration mode, (**c**) third order vibration mode, and (**d**) fourth order vibration mode.

**Figure 5:**
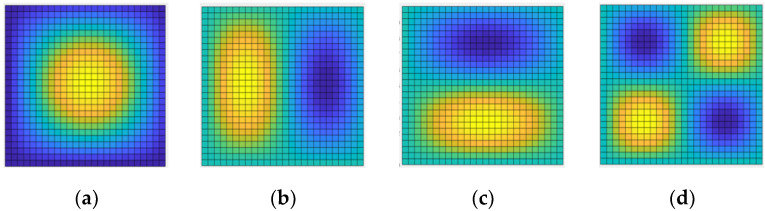
The first four-order vibration of MATLAB. (**a**) First order vibration mode, (**b**) second order vibration mode, (**c**) third order vibration mode, and (**d**) fourth order vibration mode.

It can be seen from Figure 4; Figure 5 that the first four orders are similar, and the finite element analysis verifies the accuracy of the calculation steps above. Next, the calculation results from MATLAB are directly applied to discuss the influence of the total number of layers, porous distribution mode, graphene platelets distribution, and graphene platelets’ geometric size on the vibration frequency of the porous composite plate.

## 5. Calculation and Discussion

### 5.1. Analysis of Stratified Convergence of GPL Reinforced Porous Composite Plate and the Influence of Boundary Conditions

The free vibration frequency of the composite plate with different layers and different boundary conditions when the porous distribution, with a porosity distribution A, is calculated, and the results are shown in Table 3.

By observing the influence of the total number of layers in Table 3 on the free vibration frequency of GPL reinforced porous composite plates, the optimal value can be determined, which is very important in ensuring excellent simulation accuracy and economical manufacturing efficiency at the same time. The results show that the free vibration frequency converged monotonously, with an increase in the total number of layers, and the difference was between *n* = 12 and *n* = 100, which was less than 5.0%. Therefore, *n* = 12 was used in the calculation below. When the boundary condition was CCCC, the vibration frequency of the composite plate was the largest. When the boundary condition was SSSS, the vibration frequency of the composite plate was the smallest. With the increase in the fixed edge (C), the vibration frequency of composite plate increased gradually.

### 5.2. Effect of Porosity Coefficient on Free Vibration Frequency of GPL Reinforced Porous Composite Plate

**Figure 6:**
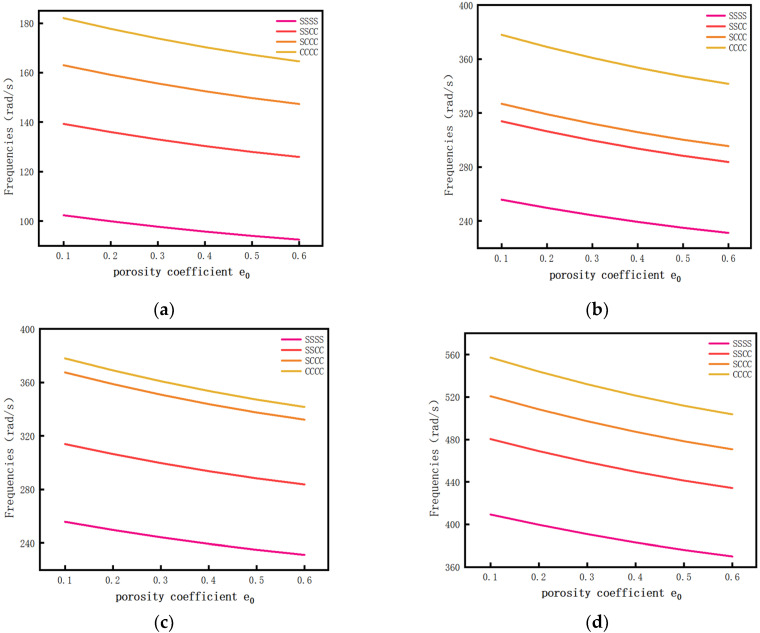
The relationship curve between the porosity coefficient and free vibration frequency. (**a**) First order vibration mode, (**b**) second order vibration mode, (**c**) third order vibration mode, and (**d**) fourth order vibration mode.

Calculate the influence of different porosity coefficients on the first four-order free vibration frequencies of the porous composite plates when there is a porosity distribution A and the GPL pattern X. Draw the result into a graph for a clearer expression, as shown in Figure 6. Specific data are shown in Table A1 of Appendix A.

It can be seen in Figure 6 that, with the increase in the porosity coefficient, the frequency of porous composite plates decreased. The increase in the internal porosity coefficient will reduce the stiffness of the porous composite plate.

### 5.3. Effect of GPL Weight Fraction on Free Vibration Frequency of GPL Reinforced Porous Composite Plate

**Figure 7:**
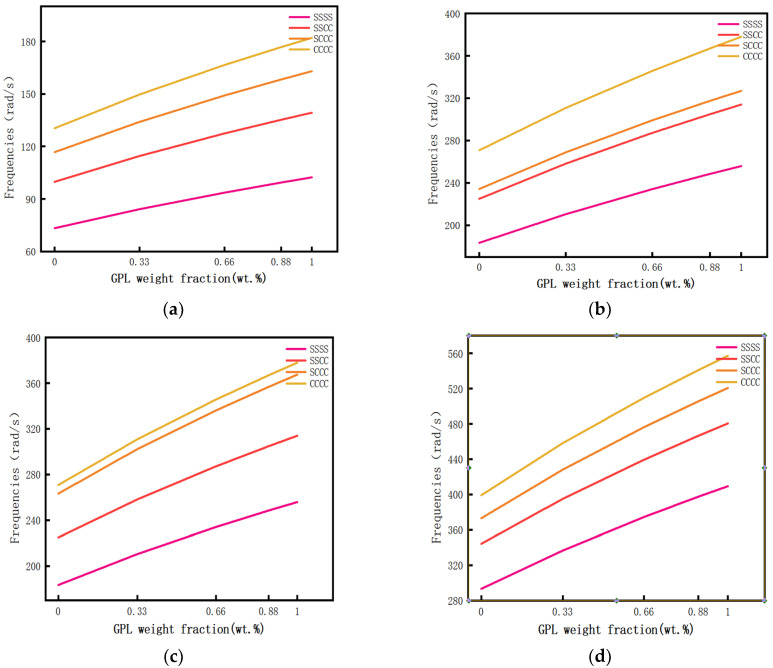
The relationship curve between GPL weight fraction and free vibration frequency. (**a**) First order vibration mode, (**b**) second order vibration mode, (**c**) third order vibration mode, and (**d**) fourth order vibration mode.

Calculate the influence of different GPL weight fractions on the first four-order free vibration frequencies of the porous composite plates when there is a porosity distribution A and the GPL pattern X. A drawn curve is shown in Figure 7. Specific data are shown in Table A2 of Appendix A.

It can be seen from Figure 7 that the frequency of the porous composite plate increases with the increase in the GPL weight fraction under other conditions unchanged. Moreover, it can be seen that a small graphene content has a great influence on the vibration frequency of the porous composite plate.

### 5.4. Effect of GPL Shape on Free Vibration Frequency of GPL Reinforced Porous Composite Plate

**Figure 8:**
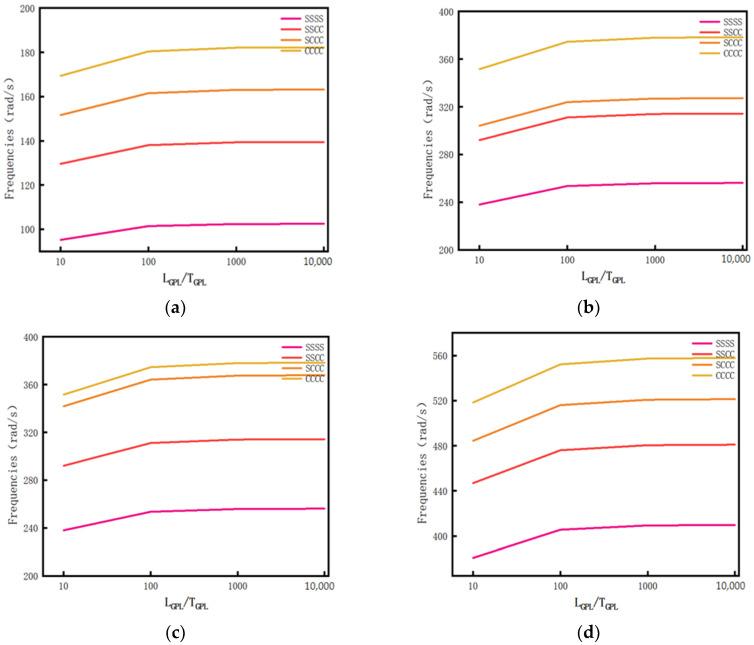
The relationship curve between *L_GPL_*/*T_GPL_* fraction and vibration frequency. (**a**) First order vibration mode, (**b**) second order vibration mode, (**c**) third order vibration mode, and (**d**) fourth order vibration mode.

Calculate the influence of different *L_GPL_*/*T_GPL_* ratios on the first four-order free vibration frequency of the porous composite plate when there is a porosity distribution A and the GPL pattern X. A drawn curve is shown in Figure 8. Specific data are shown in Table A3 of Appendix A.

Figure 8 shows that the frequency of the GPL reinforced porous composite plate increases with the increase in *L_GPL_*/*T_GPL_*. As shown in the figure, when the ratio was 10 to 100 and the boundary condition was fixed for each order, the frequency increase amplitude was obvious, and the influence on the frequency of the GPL reinforced porous composite plate was obvious. When the ratio was greater than 100, the frequency change was very small.

**Figure 9:**
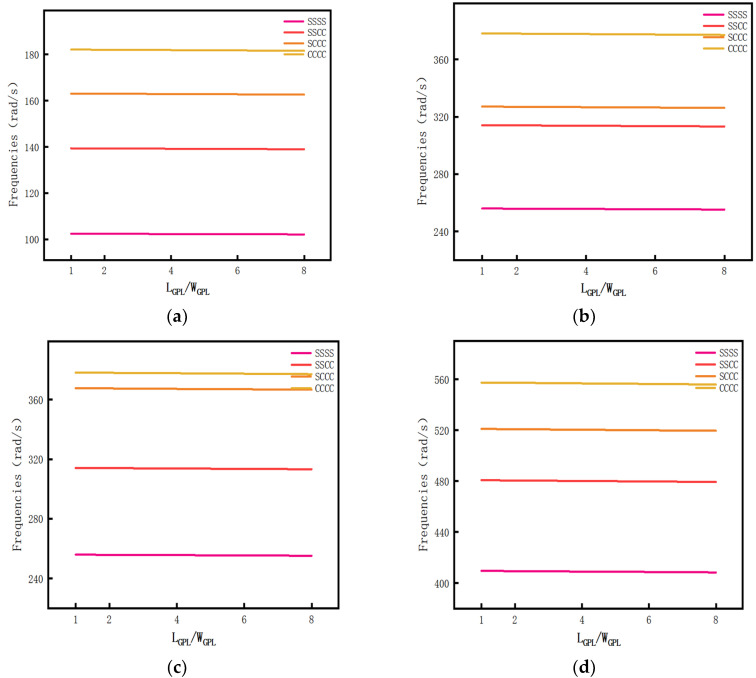
The relationship curve between *L_GPL_*/*W_GPL_* fraction and vibration frequency. (**a**) First order vibration mode, (**b**) second order vibration mode, (**c**) third order vibration mode, and (**d**) fourth order vibration mode.

Calculate the influence of different *L_GPL_*/*W_GPL_* ratio on the first four-order free vibration frequencies of GPL reinforced porous composite plate when there is a porosity distribution A and the GPL pattern X. A drawn curve is shown in Figure 9. Specific data are shown in Table A4 of Appendix A.

It can be seen in Figure 9 that, with the fixed boundary conditions of each order, the free vibration frequency of the GPL reinforced porous composite plate decreased with the increase in the ratio of *L_GPL_*/*W_GPL_*, but it had a smaller impact.

### 5.5. The Effect of Porous Distribution and GPL Distribution Pattern on the Free Vibration Frequency of GPL Reinforced Porous Composite Plate

Calculate the free vibration frequency of GPL reinforced porous composite plate with different porous distributions and GPL distribution patterns. The results are shown in Table 4.

In the case of fixed boundary conditions of each order, when the distribution mode of the GPL distribution pattern is fixed, the GPL reinforced porous composite plate free vibration frequency was the highest when the porous distribution was porosity distribution A, and the lowest when the GPL reinforced porous composite plate free vibration frequency was porosity distribution B, and the free vibration frequency of uniform porosity distribution was between them both. When the internal porous distribution of the plate was fixed, the free vibration frequency of the GPL reinforced porous composite plate was the highest, and when the GPL pattern was X, it was the lowest when the GPL pattern was O, and the free vibration frequency of the GPL reinforced porous composite plate was between them both when the GPL pattern was U. It can be concluded that the maximum free vibration frequency can be obtained by combining the porosity distribution A and with the distribution mode of the GPL pattern X.

## 6. Conclusions

The combination of porosity design and graphene reinforcement can improve the strength and stability of high value-added airplane plate structures, such as large transport aircrafts and fighters. In this work, the influences of the total number of layers, porous distribution patterns, GPL distribution patterns, porosity coefficients, GPL weight fractions, and GPL shape and boundary conditions on the free vibration frequency of GPL reinforced porous composite were studied, and a simulation analysis was conducted using finite element software in order to verify the accuracy of the conclusion. It is very important to analyze the structure of graphene reinforced composites and its application in engineering. The results show that:

1. The freer the vibration frequency of the GPL reinforced porous composite plate monotonically converged with the increase in the total number of layers, and *n* = 12 was the most suitable number of layers. With the increase in the fixed edge (C), the vibration frequency of composite plate increased gradually. When the boundary condition was CCCC, the vibration frequency of the porous composite plate at the maximum;
2. The freer the vibration frequency of the GPL reinforced porous composite plate decreased with the increase in the porosity coefficient, increased with the increase in the GPL weight fraction, increased with the increase in the *L_GPL_*/*T_GPL_*, and decreased with the increase in *L_GPL_*/*W_GPL_*. This can help us better select the size and content of graphene platelets to be applied to practice;
3. The maximum free vibration frequency can be obtained by combining the porosity distribution A and with the distribution mode of the GPL pattern X. This combination greatly verifies the properties of the reinforcement materials and the possibility of weight reduction in aircraft design, and can be applied to the aerospace industry, which can play a better role.

## Figures and Tables

**Table 1:** Porosity coefficients for different distributions.

*e*_0_	$e_{0}^{*}$	α
0.1	0.1738	0.9361
0.2	0.3442	0.8716
0.3	0.5103	0.8064
0.4	0.6708	0.7404
0.5	0.8231	0.6733
0.6	0.9612	0.6047

**Table 2:** Comparison of free vibration frequency ω (rad/s) results between MATLAB and ANSYS.

Boundary Conditions	Order	MATLAB Calculation Results	ANSYS Analysis Results
SSSS	1	102.35	102.22
	2	255.87	255.77
	3	255.87	255.77
	4	409.40	406.96
SSCC	1	139.31	140.92
	2	313.95	314.80
	3	313.95	316.02
	4	480.47	480.43
SCCC	1	163.01	166.27
	2	326.93	329.72
	3	367.50	370.15
	4	520.77	522.59
CCCC	1	182.06	188.34
	2	377.99	382.58
	3	377.99	382.58
	4	557.23	561.92

**Table 3:** The free frequencies ω (rad/s) of GPL reinforced porous composite plates—effect of total layer number and the boundary condition.

Boundary Conditions\Number of Layers	2	4	6	8	10	12	100
SSSS	107.83	103.37	102.72	102.50	102.40	102.35	102.23
SSCC	146.77	140.70	139.81	139.52	139.38	139.31	139.15
SCCC	171.74	164.64	163.60	163.25	163.09	163.01	162.82
CCCC	191.81	183.88	182.72	182.33	182.16	182.06	181.85

**Table 4:** Free vibration frequency ω (rad/s) of GPL reinforced porous composite plate with different porous distributions and GPL distribution patterns.

Boundary Conditions	Porosity A/GPL X	Porosity A/GPL U	Porosity A/GPL O	Uniform/GPL X	Uniform/GPL U	Uniform/GPL O	Porosity B/GPL X	Porosity B/GPL U	Porosity B/GPL O
SSSS	102.35	93.54	86.27	100.91	92.19	85.13	98.38	89.84	83.17
SSCC	139.31	127.32	117.43	137.35	125.48	115.87	133.91	122.28	113.20
SCCC	163.01	148.98	137.40	160.71	146.82	135.58	156.69	143.09	132.46
CCCC	182.06	166.39	153.47	179.50	163.98	151.43	175.01	159.81	147.94

## Data Availability

The data is available within the article and can be requested from the corresponding authors.

## Appendix A

**Table A1:** Free vibration frequency ω (rad/s) of GPL reinforced porous composite plate with different porosity coefficients.

Boundary Conditions	Order	*e*_0_ = 0.1	*e*_0_ = 0.2	*e*_0_ = 0.3	*e*_0_ = 0.4	*e*_0_ = 0.5	*e*_0_ = 0.6
SSSS	1	102.35	99.92	97.73	95.75	94.01	92.51
	2	255.87	249.81	244.32	239.39	235.02	231.28
	3	255.87	249.81	244.32	239.39	235.02	231.28
	4	409.40	399.70	390.91	383.02	376.03	370.05
SSCC	1	139.31	136.01	133.02	130.33	127.96	125.92
	2	313.95	306.51	299.78	293.72	288.37	283.78
	3	313.95	306.51	299.78	293.72	288.37	283.78
	4	480.47	469.09	458.78	449.51	441.32	434.29
SCCC	1	163.01	159.15	155.65	152.50	149.72	147.34
	2	326.93	319.19	312.17	305.87	300.29	295.51
	3	367.50	358.80	350.91	343.82	337.55	332.18
	4	520.77	508.43	497.26	487.21	478.33	470.72
CCCC	1	182.06	177.75	173.84	170.33	167.22	164.56
	2	377.99	369.03	360.92	353.63	347.19	341.66
	3	377.99	369.03	360.92	353.63	347.19	341.66
	4	557.23	544.03	532.07	521.33	511.82	503.68

**Table A2:** Free vibration frequency ω (rad/s) of GPL reinforced porous composite plate with different GPL weight fractions (wt.%).

Boundary Conditions	Order	wt.% = 0	wt.% = 0.33	wt.% = 0.66	wt.% = 0.88	wt.% = 1
SSSS	1	73.33	84.15	93.61	99.37	102.35
	2	183.31	210.37	234.03	248.41	255.87
	3	183.31	210.37	234.03	248.41	255.87
	4	293.30	336.60	374.45	397.46	409.40
SSCC	1	99.80	114.54	127.42	135.25	139.31
	2	224.92	258.12	287.15	304.80	313.95
	3	224.92	258.12	287.15	304.80	313.95
	4	344.22	395.03	439.46	466.46	480.47
SCCC	1	116.78	134.02	149.09	158.26	163.01
	2	234.22	268.80	299.03	317.40	326.93
	3	263.29	302.15	336.13	356.79	367.50
	4	373.09	428.17	476.32	505.59	520.77
CCCC	1	130.43	149.69	166.52	176.75	182.06
	2	270.80	310.77	345.72	366.97	377.99
	3	270.80	310.77	345.72	366.97	377.99
	4	399.21	458.15	509.67	540.99	557.23

**Table A3:** Free vibration frequency ω (rad/s) of GPL reinforced porous composite plates with different *L_GPL_*/*T_GPL_* ratios.

Boundary Conditions	Order	*L_GPL_*/*T_GPL_* = 10	*L_GPL_*/*T_GPL_* = 100	*L_GPL_*/*T_GPL_* = 1000	*L_GPL_*/*T_GPL_* = 10,000
SSSS	1	95.21	101.41	102.35	102.45
	2	238.03	253.52	255.87	256.12
	3	238.03	253.52	255.87	256.12
	4	380.85	405.63	409.40	409.79
SSCC	1	129.59	138.03	139.31	139.44
	2	292.06	311.06	313.95	314.26
	3	292.06	311.06	313.95	314.26
	4	446.97	476.05	480.47	480.94
SCCC	1	151.64	161.51	163.01	163.17
	2	304.13	323.93	326.93	327.25
	3	341.88	364.12	367.50	367.86
	4	484.45	515.98	520.77	521.27
CCCC	1	169.36	180.39	182.06	182.24
	2	351.63	374.51	377.99	378.36
	3	351.63	374.51	377.99	378.36
	4	518.38	552.11	557.23	557.77

**Table A4:** Free vibration frequency ω (rad/s) of GPL reinforced porous composite plates with different *L_GPL_*/*W_GPL_* ratios.

Boundary Conditions	Order	*L_GPL_*/*W_GPL_* = 1	*L_GPL_*/*W_GPL_* = 2	*L_GPL_*/*W_GPL_* = 4	*L_GPL_*/*W_GPL_* = 6	*L_GPL_*/*W_GPL_* = 8
SSSS	1	102.39	102.35	102.27	102.18	102.10
	2	255.98	255.87	255.66	255.46	255.25
	3	255.98	255.87	255.66	255.46	255.25
	4	409.57	409.40	409.06	408.74	408.40
SSCC	1	139.37	139.31	139.19	139.08	138.97
	2	314.08	313.95	313.70	313.45	313.19
	3	314.08	313.95	313.70	313.45	313.19
	4	480.67	480.47	480.08	479.70	479.31
SCCC	1	163.07	163.01	162.87	162.75	162.61
	2	327.07	326.93	326.66	326.41	326.14
	3	367.66	367.50	367.20	366.91	366.61
	4	520.98	520.77	520.34	519.93	519.51
CCCC	1	182.14	182.06	181.91	181.77	181.62
	2	378.14	377.99	377.68	377.38	377.07
	3	378.14	377.99	377.68	377.38	377.07
	4	557.46	557.23	556.78	556.34	555.88
